# Comprehensive Analysis of *C. glutamicum* Anaplerotic Deletion Mutants Under Defined d-Glucose Conditions

**DOI:** 10.3389/fbioe.2020.602936

**Published:** 2021-01-20

**Authors:** Jannick Kappelmann, Bianca Klein, Mathias Papenfuß, Julian Lange, Bastian Blombach, Ralf Takors, Wolfgang Wiechert, Tino Polen, Stephan Noack

**Affiliations:** ^1^Institute of Bio- and Geosciences 1, IBG-1: Biotechnology, Forschungszentrum Jülich GmbH, Jülich, Germany; ^2^Institute of Biochemical Engineering, Braunschweig University of Technology, Braunschweig, Germany; ^3^Institute of Biochemical Engineering, University of Stuttgart, Stuttgart, Germany; ^4^Microbial Biotechnology, Campus Straubing for Biotechnology and Sustainability, Technical University of Munich, Straubing, Germany

**Keywords:** *Corynebacterium glutamicum*, anaplerosis, phosphoenolpyruvate carboxylase, pyruvate carboxylase, phosphoenolpyruvate carboxykinase, oxaloacetate decarboxylase, malic enzyme, methylcitrate cycle

## Abstract

Wild-type *C. glutamicum* ATCC 13032 is known to possess two enzymes with anaplerotic (C4-directed) carboxylation activity, namely phosphoenolpyruvate carboxylase (PEPCx) and pyruvate carboxylase (PCx). On the other hand, C3-directed decarboxylation can be catalyzed by the three enzymes phosphoenolpyruvate carboxykinase (PEPCk), oxaloacetate decarboxylase (ODx), and malic enzyme (ME). The resulting high metabolic flexibility at the anaplerotic node compromises the unambigous determination of its carbon and energy flux in *C. glutamicum* wild type. To circumvent this problem we performed a comprehensive analysis of selected single or double deletion mutants in the anaplerosis of wild-type *C. glutamicum* under defined d-glucose conditions. By applying well-controlled lab-scale bioreactor experiments in combination with untargeted proteomics, quantitative metabolomics and whole-genome sequencing hitherto unknown, and sometimes counter-intuitive, genotype-phenotype relationships in these mutants could be unraveled. In comparison to the wild type the four mutants *C. glutamiucm* Δ*pyc, C. glutamiucm* Δ*pyc* Δ*odx, C. glutamiucm* Δ*ppc* Δ*pyc*, and *C. glutamiucm* Δ*pck* showed lowered specific growth rates and d-glucose uptake rates, underlining the importance of PCx and PEPCk activity for a balanced carbon and energy flux at the anaplerotic node. Most interestingly, the strain *C. glutamiucm* Δ*ppc* Δ*pyc* could be evolved to grow on d-glucose as the only source of carbon and energy, whereas this combination was previously considered lethal. The prevented anaplerotic carboxylation activity of PEPCx and PCx was found in the evolved strain to be compensated by an up-regulation of the glyoxylate shunt, potentially in combination with the 2-methylcitrate cycle.

## Introduction

*C. glutamicum* is one of the most important organisms for industrial biotechnology and the current product spectrum that is accessible with this host comprises proteinogenic as well as non-proteinogenic amino acids, organic acids, diamines, vitamins, aromates, and alcohols (Becker et al., 2018; Kogure and Inui, 2018). Most production strains have been generated by classical mutagenesis and selection, as well as by targeted and evolutionary metabolic engineering approaches (Lee and Wendisch, 2017; Stella et al., 2019). With the aim to enhance predictability of cellular functions and to reduce interference with heterologous pathways new chassis strains were introduced (Baumgart et al., 2013, 2018; Unthan et al., 2015). Several targeted and untargeted proteomics methods were developed, enabling relative, and absolute quantification of cytosolic as well as membrane-bound proteins (Fränzel et al., 2009; Voges and Noack, 2012; Trötschel et al., 2013; Küberl et al., 2014; Voges et al., 2015; Noack et al., 2017).

**Figure 1:**
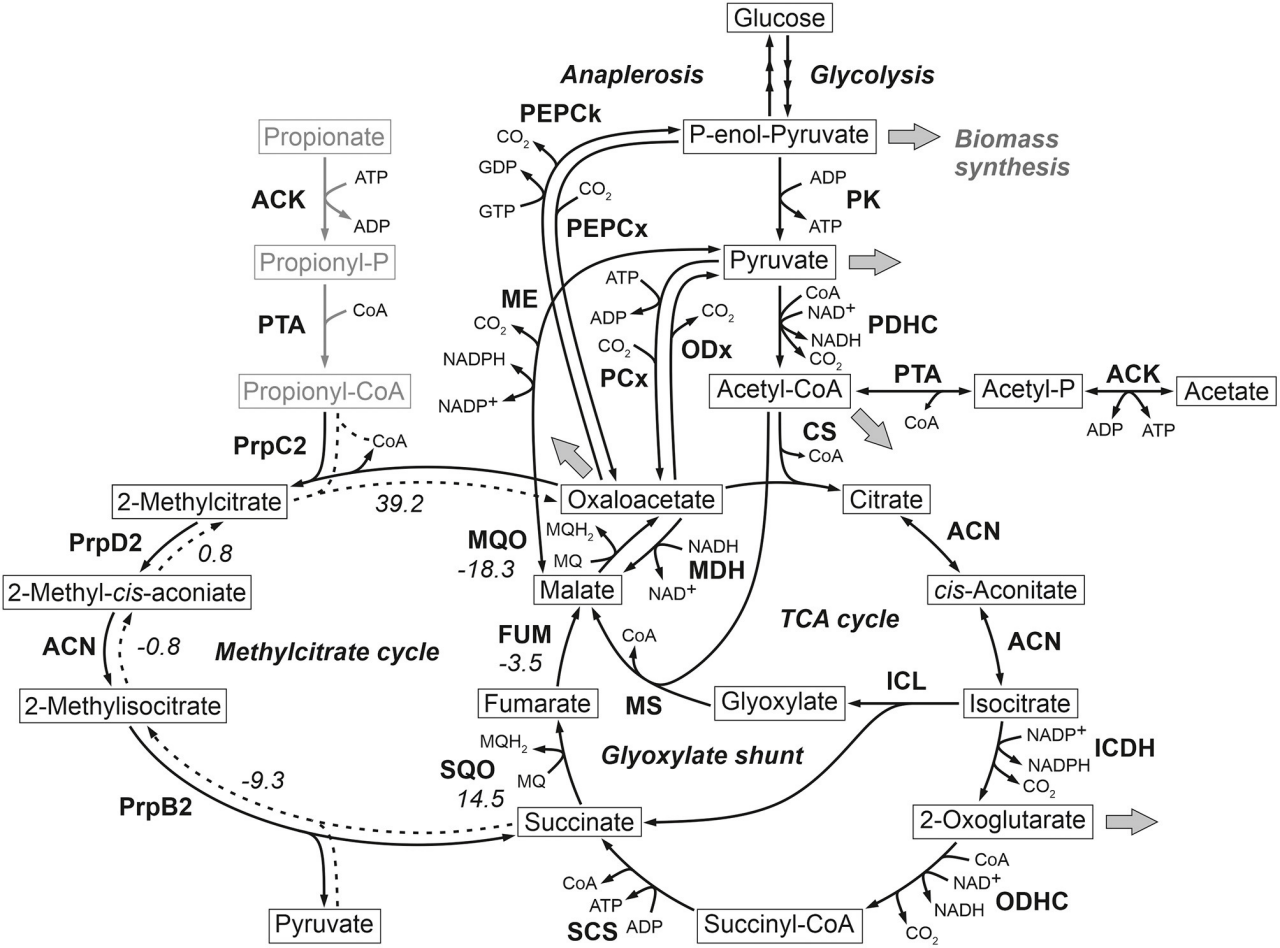
Reactions comprising the anaplerotic node, TCA cycle, glyoxylate shunt and methylcitrate cycle in *C. glutamicum* ATCC 13032. Precursor effluxes into biomass are depicted by gray arrows. For some reactions the standard Gibbs energy of reaction in kJ/mol are shown, which were estimated using the eQuilibrator tool (Flamholz et al., 2012). The values for the methylcitrate cycle refer to the assumed reverse reaction direction represented by broken lines. ACN, aconitase; ACK, acetate kinase; CS, citrate synthase; FUM, fumarase; ICDH, isocitrate dehydrogenase; ICL, isocitrate lyase; MDH, malate dehydrogenase; ME, malic enzyme; MQO, malate: quinone oxidoreductase; MS, malate synthase; ODHC, 2-oxoglutarate dehydrogenase complex; ODx, oxaloacetate decarboxylase; PCx, pyruvate carboxylase; PDHC, pyruvate dehydrogenase complex; PEPCk, phosphoenolpyruvate carboxykinase; PEPCx, phosphoenolpyruvate carboxylase; PPS, phosphoenolpyruvate synthetase; PK, pyruvate kinase; PQO, pyruvate: quinone oxidoreductase; PrpB2, 2-methylcitrate lyase; PrpC2, 2-methylcitrate synthase; PrpD2, 2-methylcitrate dehydratase; PTA, phosphotransacetylase; SCS, succinyl-CoA-synthetase; SQO, succinate: quinone oxidoreductase.

At the phosphoenolpyruvate-pyruvate-oxaloacetate node *C. glutamicum* ATCC 13032 (wild type) is known to possess two enzymes with anaplerotic (C4-directed) carboxylation activity, namely phosphoenolpyruvate carboxylase (PEPCx) and pyruvate carboxylase (PCx). On the other hand, C3-directed decarboxylation can be catalyzed by the three enzymes phosphoenolpyruvate carboxykinase (PEPCk), oxaloacetate decarboxylase (ODx), and malic enzyme (ME). While all enzymes show *in vitro* activity in cells grown in defined d-glucose media (Cocaign-Bousquet et al., 1996; Uy et al., 1999; Klaffl and Eikmanns, 2010; Blombach et al., 2013), only PEPCx and PCx are currently considered as dependent essential anaplerotic enzymes under these conditions (Figure 1).

Recently, the anaplerotic node of *C. glutamicum*, which represents a very flexible knot for diverting the carbon and energy flux introduced by different potential substrates, has again attracted our attention. Following detailed mathematical modeling and computational analyses, we could prove that only certain anaplerotic deletion mutants allow to uniquely determine the anaplerotic fluxes (Kappelmann et al., 2016).

Following shake flask experiments, it was shown that a single inactivation of either PEPCx, PCx, ODx or ME in wild-type *C. glutamicum* has no effect on biomass growth (Peters-Wendisch et al., 1993, 1998; Gourdon et al., 2000; Klaffl and Eikmanns, 2010). The only exception was found for PEPCk, whose inactivation resulted in a lower growth rate (Riedel et al., 2001). In contrast, the combined removal of PEPCx and PCx is thought to be lethal for *C. glutamicum* when grown under glycolytic conditions as no other carboxylation reaction enables replenishment of tricarboxylic acid intermediates (Peters-Wendisch et al., 1998). Recently, Schwentner et al. were able to evolve *C. glutamicum* Δ*ppc* Δ*pyc* to grow on CGXII medium with 20 g L^−1^ d-glucose and 1 g L^−1^ yeast extract (or alternatively 1 mM l-glutamate) (Schwentner et al., 2018). Comparative whole-genome sequencing revealed isocitrate dehydrogenase (ICD) as consistent target and the identified mutations could be linked to diminished ICD activities as well as increased activities of the glyoxylate shunt enzymes isocitrate lyase (ICL) and malate synthase (MS). Operation of the glyoxylate shunt can substitute for the missing carboxylation reactions to replenish oxaloacetate required for growth. Interestingly, on pure d-glucose media this evolution failed in the study reported (Schwentner et al., 2018).

In our study, we performed a comprehensive analysis of selected single or double deletion mutants in the anaplerosis of wild-type *C. glutamicum* under defined d-glucose conditions without other carbon supplements. By applying well-controlled lab-scale bioreactor experiments in combination with untargeted proteomics, quantitative metabolomics and whole-genome sequencing hitherto unknown genotype-phenotype relationships in these mutants could be unraveled and these are discussed in detail with regard to published data.

## Materials and Methods

### Bacterial Strains

All strains, plasmids and oligonucleotids used in this study are listed in Table 1. The *C. glutamicum* WT as well as the single deletion mutants Δ*pyc*, Δ*malE*, Δ*pck* are from Blombach et al. (2013). The double deletion mutant Δ*ppc* Δ*pyc* is described in Schwentner et al. (2018). The three remaining double deletion mutants Δ*pyc* Δ*odx*, Δ*ppc* Δ*malE* and Δ*pck* Δ*malE* were constructed by chromosomal inactivation of the ODx gene *odx* in *C. glutamicum* Δ*pyc*, as well as the ME gene *malE* in *C. glutamicum* Δ*pck* and *C. glutamicum* Δ*ppc* using the plasmids pK19mobsacB-Δ*odx* and pK19mobsacB-Δ*malE*. Isolation of plasmids from *Escherichia coli* was performed as described elsewhere (Eikmanns et al., 1994). Plasmid DNA transfer into *C. glutamicum* was carried out by electroporation and recombinant strains were selected on Luria–Bertani Brain Heart Infusion agar plates containing appropriate concentrations of kanamycin (50 μg mL^−1^) (Van Der Rest et al., 1999). The replacement at the chromosomal locus was verified by colony-PCR using primers odxfow/odxrev and Co-malE1/Co-malE1, respectively (Klaffl and Eikmanns, 2010; Blombach et al., 2011). Subsequently, shaking flasks with CGXII preculture medium containing 1% d-glucose (w v^−1^) were inoculated with the corresponding strains (Unthan et al., 2014). Each culture was harvested during the late exponential phase following centrifugation at 4,500 rpm for 10 min, resuspension in sterile saline and finally in 20% (v v^−1^) glycerol solution in sterile 0.9% (w v^−1^) NaCl in distilled water. From this solution cryo stocks were prepared and immediately stored at −80°C. The Δ*ppc* Δ*pyc* mutant was able to grow on acetate, from which a corresponding cryo-culture was produced.

**Table 1:** Strains, plasmids, and oligonucleotides used in this study.

**Strains**	**Relevant characteristic(s) or sequence**	**Source/reference or purpose**
*C. glutamicum* WT	wild type (WT) strain ATCC 13032, biotin-auxotrophic	American Type Culture Collection
*C. glutamicum* Δ*pyc*	*C. glutamicum* WT with deletion of the *pyc* gene encoding pyruvate carboxylase	Blombach et al., 2013
*C. glutamicum* Δ*malE*	*C. glutamicum* WT with deletion of the *malE* gene encoding malic enzyme	Blombach et al., 2013
*C. glutamicum* Δ*pck*	*C. glutamicum* WT with deletion of the *pck* gene encoding phosphoenolpyruvate carboxykinase	Blombach et al., 2013
*C. glutamicum* Δ*ppc* Δ*pyc*	*C. glutamicum* WT with deletion of the *ppc* and *pyc* gene encoding phosphoenolpyruvate carboxylase and pyruvate carboxylase, respectively	Schwentner et al., 2018
*C. glutamicum* Δ*pck* Δ*malE*	*C. glutamicum* WT with deletion of the *pck* and *malE* gene encoding phosphoenolpyruvate carboxykinase and malic enzyme, respectively	This work
*C. glutamicum* Δ*pyc* Δ*odx*	*C. glutamicum* WT with deletion of the *pyc* and *odx* gene encoding pyruvate carboxylase and oxaloacetate decarboxylase, respectively	This work
*C. glutamicum* Δ*ppc* Δ*malE*	*C. glutamicum* WT with deletion of the *ppc* and *malE* gene encoding phosphoenolpyruvate carboxylase and malic enzyme, respectively	This work
**Plasmids**		
pK19*mobsacB*-Δ*malE*	pK19*mobsacB* carrying a truncated *malE* gene	Blombach et al., 2011
pK19*mobsacB*-Δ*odx*	pK19*mobsacB* carrying the *odx* gene with internal 679-bp deletion	Klaffl and Eikmanns, 2010
**Oligonucleotides**		
odxfow	5′-ACCGGCATCAAATTGTGTC-3′	Primer to verify deletion of *odx*
odxrev	5′-TTGCCTTGAGCACAATGTC-3′	Primer to verify deletion of *odx*
Co-malE1	5′- CTTCCAGACACGGAATCAGAG-3′	Primer to verify deletion of *malE* (Blombach et al., 2011)
Co-malE2	5′- GTGATCCTTCCGAGCGTTCC-3′	Primer to verify deletion of *malE* (Blombach et al., 2011)

### Evolution and Whole-Genome Sequencing of *C. glutamicum* Δ*ppc* Δ*pyc*

*C. glutamicum* Δ*ppc* Δ*pyc* was grown in microtiter plates in a BioLector parallel cultivation system (m2p-labs). Flowerplates with optodes for optical pH and dissolved oxygen (DO) measurements were employed. A cryo-culture for inoculation was washed once with sterile saline and resuspended in d-glucose-free CGXII medium. From this inoculum 50 μL were transferred into each well containing 950 μL 1% d-glucose medium. The plates were sterilely sealed and incubated at 30°C at 1,300 rpm.

For whole-genome sequencing, 200 μL of a well-inoculated at OD_init_ = 1 (see Supplementary Figure 1) and after growth has ceased was used to inoculate a subsequent shaking flask culture, from which a cryo-culture was produced. From this cryo-culture a sample was generated for whole-genome sequencing using the Illumina platform followed by sequence analysis as described elsewhere (Kranz et al., 2017).

### Bioreactor Cultivations

*C. glutamicum* deletion strains were cultivated in a DASGIP parallel fermentation system (Eppendorf). Bioreactor cultivations of *C. glutamicum* strains were carried out with defined CGXII medium containing 1% d-glucose and 0.1% undiluted Antifoam 204 but no 3-(N-Morpholino)propanesulfonic acid (MOPS) buffer (Unthan et al., 2014). Bioreactors were inoculated from a preculture in CGXII medium buffered with 42 g L^−1^ MOPS at pH 7 which was inoculated directly from a cryo-culture of each strain in 80% 0.9% NaCl/20% glycerol (v v^−1^) stored at −80°C. During bioreactor cultivations DO levels were maintained above 30% by adjusting stirrer speed and oxygen content of the inlet air. The gassing rate was set to 1 vvm and the pH was maintained at pH 7 by feeding either 4 M NaOH or 4 M HCl. The cultivation temperature was 30°C.

For quantitative metabolomics, samples from bioreactor cultivations were drawn into a syringe in technical duplicate at two time points yielding a total of four technical replicates per strain. These time points correspond to target BV concentrations of 5 μL mL^−1^ (OD_600_ = 6.3) and 10 μL mL^−1^ (OD_600_ = 12.5), covering the mid-exponential phase (cmp. Supplementary Figure 2). The actual BV concentration in each sample was measured after sampling and used to calculate the extraction volume.

For untargeted proteomics, sampling was performed directly after all quenching samples for the metabolome analysis had been taken. From each reactor samples were drawn in technical quintuplicate by centrifuging 10 mL of culture broth for each replicate (10 min, 4500 rpm, GS-15R Centrifuge, Beckman Coulter). After the supernatant was decanted, the biomass pellets were immediately placed in aluminum racks at −20°C.

For biovolume (BV) measurements, cultivation samples were diluted 1:200 or 1:2,000 depending on the biomass concentration in 10 mL CASYton buffer (OMNI Life Science GmbH). The size distribution of the sample was determined by the MultiSizer3 Coulter Counter (Beckman Coulter) and the biovolume was computed by calculating the first moment of the distribution, assuming a spherical shape of the measured cells. Cell dry weight (CDW) was determined by centrifuging 2 mL of a bioreactor sample in a pre-dried and pre-weighted Eppendorf tube at 13,000 rpm for 7 min. The cells were washed in 1 mL 0.9% (w v^−1^) NaCl by resuspension and renewed centrifugation. After decanting the supernatant, the pellets were dried for at least 2 days at 80°C.

A correlation between BV in μL mL^−1^ and CDW in g L^−1^ was derived for the Δ*pyc* Δ*odx*, Δ*ppc* Δ*malE* and WT strain (see Supplementary Figure 3) and then applied to calculate the CDW for all cultivations assuming a standard deviation of 5%.

### Estimation of Extracellular Rates

Specific rates for biomass growth (μ) and d-glucose uptake (π_*GLC*_) were estimated using a model-based approach and process data from the exponential phases of corresponding batch cultivations. In short, the remaining d-glucose concentration *c*_*GLC*_(*t*) at any given time point *t* is the integral over the volumetric uptake rate π_*GLC,vol*_(*t*) given in mmol L^−1^ h^−1^:

(1) $$\begin{array}{l} c_{GLC}\left(t\right)=c_{GLC,0}-\int_{t_{0}}^{t}\pi_{GLC,vol}\left(t\right)dt \end{array}$$

Assuming a constant d-glucose uptake from exponentially growing cells, it holds:

(2) $$\begin{array}{l} \pi_{GLC,vol}\left(t\right)=\pi_{GLC}\cdot X\left(t\right) \end{array}$$

where $X\left(t\right)=X_{0}\cdot e^{\mu \cdot t}$ denotes the biomass concentration in μL mL^−1^ or g L^−1^, depending on whether the biomass signal is BV or CDW. Inserting Equations (2) into (1) and carrying out the integration yields:

(3) $$\begin{array}{l} c_{GLC}\left(t\right)=C-\frac{\pi_{GLC}}{\mu }{\cdot X}_{0}\cdot e^{\mu \cdot t} \end{array}$$

with model parameters *X*_0_, μ, π_*GLC*_, and *C*, where the latter absorbs the integration constant and the initial substrate concentration. Equations (2) and (3) were jointly fitted to the experimentally observed time courses of biomass and substrate concentration, respectively. The end of the exponential phase was judged by the peak in CO_2_ volume fraction in the exhaust gas stream (see Supplementary Figure 2).

The fitting procedure was carried out using a sequential quadratic programming optimization routine from MATLAB (Mathworks Inc., R2019b). The estimation of confidence intervals was based on a parametric Monte Carlo bootstrapping approach from literature (Dalman et al., 2013). In short, every available measurement was perturbed independently within the normal distribution described by the measurement value and its standard deviation of the respective measurement. The perturbation operation consisted in sampling from the normal distribution of each measurement point and inserting the sample as measurements. The above-described variance-weighted least-square fit was then re-performed. The sample was generated as a Latin-Hypercube sample using the lhs-function of MATLAB and comprised 1,000 samples if not otherwise stated. The upper and lower confidence bounds were then derived as the α-th and (1-α)-th percentile of the parameter sample obtained from 1000 least-squared fits. If not otherwise stated α is set to 0.25, the confidence interval being the Interquartile Range (IQR).

The total CO_2_ formation rate π_*CO*2,*tot*_(*t*) given in mol h^−1^ was calculated from balancing the gas phase of the bioreactor as:

(4) $$\begin{array}{l} \pi_{CO_{2},tot}\left(t\right)\\ =\frac{F\cdot p}{R\cdot T}\left[\frac{100-\Phi_{O_{2}}^{\alpha }-\Phi_{CO_{2}}^{\alpha }}{100-\Phi_{O_{2}}^{\omega }\left(t\right)-\Phi_{CO_{2}}^{\omega }\left(t\right)}\cdot \frac{\Phi_{CO_{2}}^{\omega }\left(t\right)}{100}-\frac{\Phi_{CO_{2}}^{\alpha }}{100}\right] \end{array}$$

where Φ denotes the volume fraction of the gas species in question in vol% and the superscripts α and ω denote the inlet and outlet concentrations, respectively. *F* denotes the inlet air flow in m^3^ h^−1^. The biomass-specific carbon dioxide formation rate π_*CO*_2__(*t*) given in mmol ${\text{g}}_{\text{CDW}}^{-1}$ h^−1^ or mmol ${\text{mL}}_{\text{BV}}^{-1}$ h^−1^, respectively, was obtained by dividing π_*CO*_2_,*tot*_(*t*) by *X*(*t*) at time point *t*.

The complete set of extracellular rate estimates for all independent bioreactor cultivation experiments can be found in Supplementary Table 1.

### Quantitative Metabolomics

The metabolome samples of all strains were spiked with identical internal standard and were measured in one acquisition batch on the LC-ESI-QqQ MS system. Organic acids and unstable sugar phosphates were measured within 24 h from the extraction of samples. Quenching, cell separation, cell extraction, isotope dilution mass spectrometry and metabolite leakage correction were performed according to previous protocols (Paczia et al., 2012; Tillack et al., 2012).

For quantification of organic acids, samples were separated using a synergy hydro C18 reversed phase column (Phenomenex) on an Agilent 1200 chromatography system (Agilent Technologies). The HPLC column outlet was coupled to a QqQ MS device (API 4000, AB Sciex) equipped with a TurboSpray ion source in negative ionization mode. The elution was isocratic at 84% buffer A and 16% buffer B at a flowrate of 0.45 mL min^−1^ at 20°C. The eluents were as follows: Buffer A: 10 mM tributylamine, 15 mM acetic acid, pH 4.95; Buffer B: methanol. MS parameters were as follows: CAD (collision gas pressure): 5, CUR (curtain gas flow): 30, GS1 (nebulizer gas flow): 70, GS2 (turbo heater gas flow): 70, IS (electrospray voltage): −4,500 V, TEM (heater gas temperature): 650°C, entrance potential: −10 eV. Injection volume was 10 μL.

For quantification of amino acids, samples were separated using Luna SCX cation exchange column at 60°C on a JASCO HPLC system. The following buffers were employed: Buffer A: 5% acetic acid, B: 15 mM ammonium acetate. The applied elution gradient can be found in Supplementary Table 2. Injection volume was 10 μL.

For quantification of sugar and nucleoside phosphates, samples were separated on a synergy hydro C18 reversed phase column at 40°C. Eluent were Buffer A: 10 mM tributylamine, 15 mM acetic acid, pH 4.95 and Buffer B: methanol. The HPLC system, the QqQ MS device and its MS parameter settings were the same as for the LC-MS/MS method for organic acids. For quantification the gradient of Supplementary Table 3 was applied. Injection volume was 10 μL.

### Generation of Ion Libraries for Proteomics

To populate our *C. glutamicum* ion library, separate IDA acquisitions of samples of *C. glutamicum* WT cultivated as described above but with different carbon sources were performed. In each cultivation the carbon source was either 55 mM d-glucose, 111 mM sodium pyruvate, 83 mM disodium-L-malate, 55 mM sodium citrate or 48 mM sodium benzoate. Each sample was lysed and 100 μg protein thereof digested and processed as described elsewhere (Voges and Noack, 2012).

These samples were separated on a Agilent 1260 Infinity HPLC system (Agilent Technologies) equipped with a 150 ^*^ 2.1 mm Ascentis Express Peptide ES-C18 column with 2.7 μm particle size and an appropriate 5 ^*^ 0.3 mm Acclaim PepMap Trap Cartridge (Thermo Scientific) which were both maintained at 25°C and a flow rate of 200 μL min^−1^. For LC separation 0.1% formic acid in LC-MS grade water (v v^−1^) was used as buffer A, whereas buffer B was 0.1% formic acid in LC-MS grade acetonitrile (v v^−1^). Before each injection, the column was equilibrated for 12 min at 97% A. After 20 μL were injected, the gradient of Supplementary Table 4 was applied. The LC-eluent was coupled to an ESI-QqTOF MS (TripleTOF 6600, AB Sciex) equipped with an DuoSpray ion source. The data acquisition was performed using Analyst TF 1.8 (AB Sciex). An information-dependent acquisition was performed on each injection during which all ions with *m/z* >300, charge state 2–4 and above intensity of 150 were selected for fragmentation.

The acquired MS2 spectra of the *C. glutamicum* digests were searched against a FASTA database of *C. glutamicum* ATCC 13032 (GenBank assembly accession: GCA_000196335.1) using ProteinPilot software 5.0 (AB Sciex) employing default probabilities for biological modifications. The confidently identified peptides of each injection were assembled into a library covering 1727 ORFs. This library incorporates the peptide confidence after identification, peptide (precursor) intensity in the MS1 scan from the IDA acquisition, fragment ion intensities and the observed peptide retention time.

### SWATH Acquisition

Starting from an IDA acquisition of each organism variable SWATH windows were calculated using the SWATH Variable Window Calculator 1.0 (AB Sciex). Using these windows, a SWATH acquisition method was set up, which employed the same chromatographic gradient and ion source setting as the IDA acquisition. The window width and CE ramp parameters can be found in Supplementary Table 5. For SWATH acquisition a digest of each sample was prepared according to the protocol of Voges and Noack, which involves mixing 50 μg unlabeled sample protein and 50 μg internal standard from a separate cultivation of *C. glutamicum* with (^15^NH_4_)_2_SO_4_ (Voges and Noack, 2012). Ten microliter of each sample was injected.

### SWATH MS Data Processing

SWATH data processing was performed using the MS/MS^all^ SWATH Acquisition MicroApp in PeakView 2.2 (AB Sciex). The ion library from above was imported from ProteinPilot into this app by excluding shared peptides but not modified ones. From the imported ion library the ten most intense peptides were selected for quantification provided they had a peptide confidence of >96%. The intensity selection is based on the MS1 survey scan intensity of each peptide in the IDA runs used to build the ion library. If <10 peptides fulfilled the above criterion for a protein, only the available peptides were quantified.

For each peptide group the 12 most intense fragment ion traces were chosen by the SWATH processing algorithm. This algorithm favors the most intense fragment ion traces from the library spectrum whose *m/z* value lies above the Q1 window of their precursor ion. For each fragment ion, within 5 min around the expected retention time, an unlabeled mass trace was extracted from the SWATH spectra within ±15 ppm of its monoisotopic mass, whereas the labeled mass trace was extracted within ±15 ppm of its fully ^15^N-labeled isotopologue. All transitions of one peptide were assembled into a so called peak group which was scored for congruency with the ion library. The false discovery rate was set to 0.1%. The finished processing session was saved as MarkerView file (.mrkvw extension), which was opened in MarkerView 1.3.1 (AB Sciex) for estimation of fold-changes (ratio of means) of protein levels between mutant and control.

### Elemental Analysis of Biomass

The concentration of carbon, nitrogen and sulfur in biomass were determined at the central Analytical core facility of Forschungszentrum Jülich (ZEA-3). Biomass samples from the early stationary phase were processed following the same procedure as for CDW content determination. The dried biomass pellet was ground to a fine powder in a pre-dried mortar using a pre-dried pestle and sent in a sealed container to ZEA-3.

## Results and Discussion

### Growth Phenotyping of Anaplerotic Deletions Mutants Under Defined d-Glucose Conditions

In our previous flux identifiability analysis focusing on the anaplerotic node in *C. glutamicum* those metabolic network structures were identified that are structurally identifiable under defined d-glucose conditions (Kappelmann et al., 2016). These structures are based on deletions of specific genes encoding for anaplerotic reactions. In this study, we comprehensively analyzed a set of mutant strains for which a unique solution using ^13^C-MFA theoretically exists as well as mutants that are non-flux-identifiable strains.

All selected deletion mutants were able to grow on defined CGXII medium with d-glucose as sole carbon and energy source, except for strain *C. glutamicum* Δ*ppc* Δ*pyc* that is deficient in PEPCx and PCx activity (Table 1). While this mutant was able to grow on acetate without lag-phase no biomass formation could be monitored within 48 h cultivation on d-glucose and this observation is concordant with Peters-Wendisch et al. (1998). However, prolonged incubation of *C. glutamicum* Δ*ppc* Δ*pyc* under defined d-glucose conditions in a microbioreactor setup and at different inoculum sizes always resulted in the onset of growth after 75 h or later, mainly after 100 h (Supplementary Figure 1).

**Figure 2:**
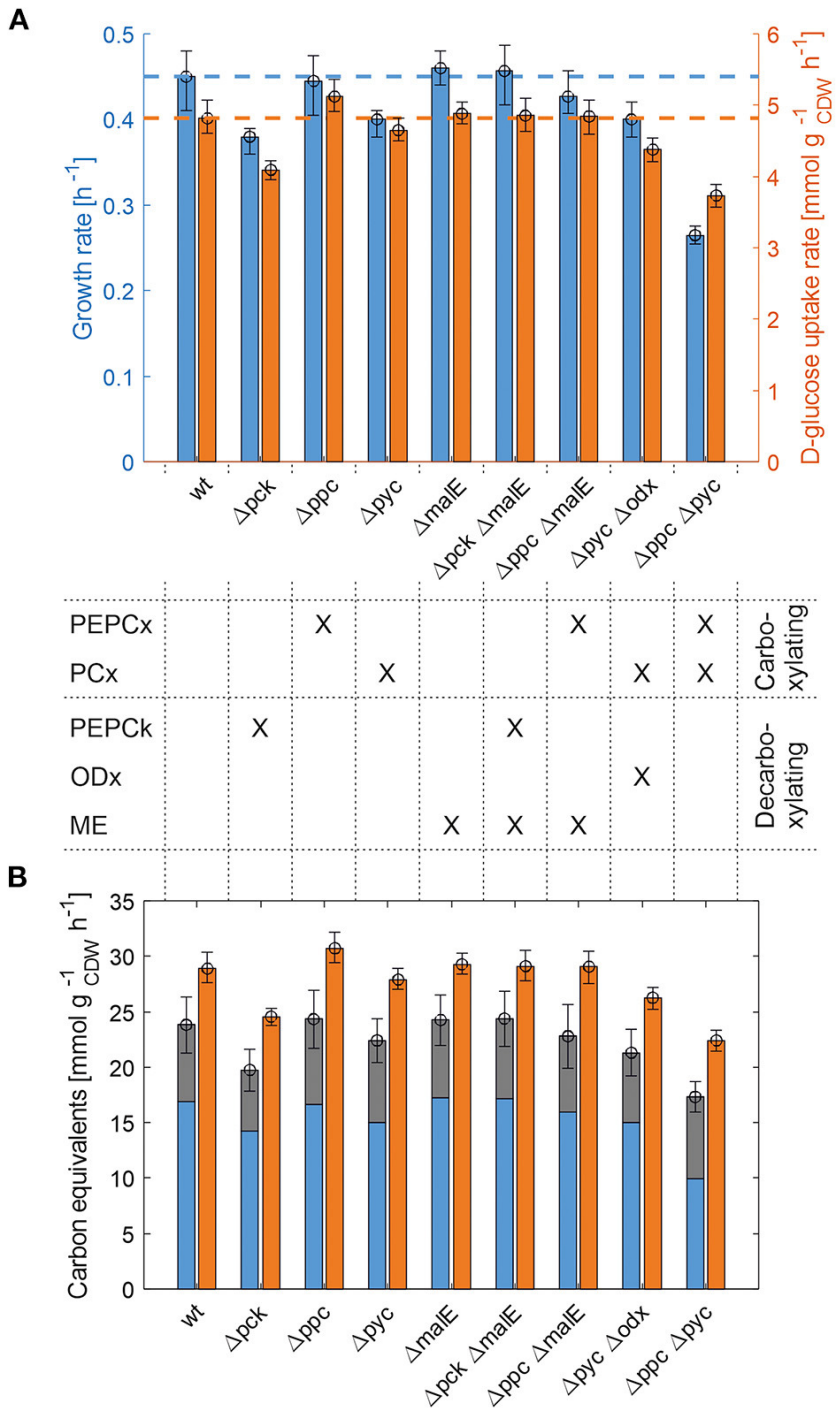
Growth phenotyping of anaplerotic deletions mutants under defined d-glucose conditions. **(A)** Estimated specific growth and d-glucose uptake rates. Mean values of rate estimates are from biological duplicate or triplicate cultivations. Error estimates are given as the minimum of the lower bound and the maximum of the upper bound of each parameter over all replicate cultivations of the mutant in question (see Supplementary Table 1). **(B)** Carbon equivalents of the specific growth rate (blue), CO_2_-formation rate (gray) and the substrate uptake rate (orange). The error bar on the stacked bars is the error on the sum of both rates, which was estimated according to Gaussian error propagation.

The whole mutant library was then cultivated under controlled bioreactor conditions on defined CGXII medium with 1% d-glucose. Specific growth and substrate uptake rates were estimated using a model-based approach (see Materials and Methods section). The d-glucose uptake rate of 4.82 mmol ${\text{g}}_{\text{CDW}}^{-1}{\text{h}}^{-1}$ and the specific growth rate of 0.45 h^−1^ for *C. glutamicum* WT agree well with literature (Buchholz et al., 2014). Based on the determined confidence intervals discernible growth phenotypes can be identified among the set of deletion mutants (Figure 2A and Table 2).

**Table 2:** Estimated specific growth rates (μ), d-glucose consumption rates (π_**GLC**_), CO_2_ formation rates (π_**CO2**_), and instantaneous carbon balance (θ) for anaplerotic deletion mutants during exponential growth.

**Strain**	**μ [h^**−1**^]**	**π_GLC_ [mmol ${\text{g}}_{\text{CDW}}^{-1}$ h^**−1**^]**	**π_CO2_ [mmol ${\text{g}}_{\text{CDW}}^{-1}$ h^**−1**^]**	**θ [–]**	**Conditions**	**References**
*C. glutamicum* ATCC 13032	0.44	–	–	–	Shake flask	Riedel et al., 2001
	0.40^a^	–	–	–	Bioreactor	Blombach et al., 2013
	0.45 ± 0.04	4.82 ± 0.25	6.94 ± 0.71	0.82 ± 0.089	Bioreactor	This work
*C. glutamicum* Δ*pck*	0.40	–	–	–	Shake flask	Riedel et al., 2001
	0.30^a^	–	–	–	Bioreactor	Blombach et al., 2013
	0.38 ± 0.02	4.10 ± 0.14	5.51 ± 0.53	0.81 ± 0.077	Bioreactor	This work
*C. glutamicum* Δ*ppc*	Equal to WT	–	–	–	Shake flask	Peters-Wendisch et al., 1993, 1996
	0.34^a^	–	–	–	Bioreactor	Blombach et al., 2013
	0.45 ± 0.04	5.12 ± 0.24	7.64 ± 1.09	0.79 ± 0.083	Bioreactor	This work
*C. glutamicum* Δ*pyc*	Nearly WT	–	–	–	Shake flask	Peters-Wendisch et al., 1998
	0.30^a^	–	–	–	Bioreactor	Blombach et al., 2013
	0.40 ± 0.02	4.65 ± 0.17	7.43 ± 0.76	0.80 ± 0.072	Bioreactor	This work
*C. glutamicum* Δ*malE*	Equal to WT	–	–	–	Shake flask	Gourdon et al., 2000
	0.34^a^	–	–	–	Bioreactor	Blombach et al., 2013
	0.46 ± 0.02	4.88 ± 0.17	7.00 ± 1.00	0.84 ± 0.080	Bioreactor	This work
*C. glutamicum* Δ*pck* Δ*malE*	0.46 ± 0.04	4.85 ± 0.24	7.27 ± 0.83	0.83 ± 0.087	Bioreactor	This work
*C. glutamicum* Δ*ppc* Δ*malE*	0.43 ± 0.03	4.84 ± 0.25	6.79 ± 1.92	0.79 ± 0.088	Bioreactor	This work
*C. glutamicum* Δ*pyc* Δ*odx*	0.40 ± 0.02	4.38 ± 0.17	6.31 ± 0.99	0.81 ± 0.078	Bioreactor	This work
*C. glutamicum* Δ*ppc* Δ*pyc*^b^	No growth	–	–	–	Shake flask	Peters-Wendisch et al., 1998
	0.27 ± 0.01	3.74 ± 0.16	7.40 ± 0.68	0.78 ± 0.070	Bioreactor	This work

*Comparison of own data with available literature data for cultivation experiments with the specified strains on defined CGXII media and d-glucose as sole carbon and energy source. Data from C. glutamicum ATCC 13032 (WT) was only included from studies with corresponding deletion mutants. In case no quantitative data was available a qualitative comparison was made*.

a *Aeration was limited to 0.1 vvm and the media contained no initial protocatechuic acid*.

b *Evolved strain after prolonged cultivation in CGXII media with d-glucose as sole carbon and energy source*.

First, the *C. glutamicum* Δ*pck* mutant showed a lower growth rate although PEPCk catalyzes a gluconeogenetic reaction, not needed under glycolytic conditions to supply biomass precursors. However, the observed growth defect clearly indicates its activity under glycolytic conditions. This result was also obtained in the study of Riedel et al. with the comparable genotype (Table 2) and in the study of Petersen et al. (2001), who inferred PEPCk activity in a *C. glutamicum* l-lysine producer from ^13^C-labeling data. Since the carbon flow catalyzed by PEPCk is of opposite direction compared to the overall carbon flow under glycolytic conditions, its catalytic effect must be of indirect nature. Strangely, the double deletion mutant *C. glutamicum* Δ*pck* Δ*malE* showed a restored growth phenotype, with a growth rate equivalent to the WT.

Deletion mutants comprising a deletion in PEPCx do not show a significantly altered growth phenotype as long as PCx is still active (Figure 2A and Table 2). The stoichiometry of the reaction catalyzed by PEPCx is identical to the reaction sequence of pyruvate kinase (PK) and PCx, which apparently fully compensates for the missing carboxylation activity of PEPCx. On the other hand, mutant strains *C. glutamicum* Δ*pyc* and *C. glutamicum* Δ*pyc* Δ*odx* do exhibit a growth phenotype. This finding underscores the role of PCx as most important anaplerotic reaction under aerobic conditions and suggests that its catalyzed flux is most likely greater than that of PEPCx in the WT under standard d-glucose conditions. Noteworthy, a *C. glutamicum* mutant with single deletion of the *odx* gene was shown to grow equally well as the wild type (Klaffl and Eikmanns, 2010).

The evolved *C. glutamicum* Δ*ppc* Δ*pyc* strain, missing both carboxylation activities shows a greatly reduced growth rate of 0.27 h^−1^ (Figure 2A and Table 2). In the absence of both PEPCx and PCx, three other and different anaplerotic activities can possibly substitute the anaplerotic activity of PEPCx and PCx: First, ME may catalyze the carboxylation of pyruvate to malate in an NADPH-dependent manner. This appears plausible since ME was found to catalyze this reaction sequence in *in vitro* assays (Cocaign-Bousquet et al., 1996; Gourdon et al., 2000). Moreover, it could be experimentally shown that a NADPH-dependent ME from *E. coli* can act as sole anaplerotic enzyme in *Saccharomyces cerevisiae* sustaining a growth rate of 0.06 h^−1^ (Zelle et al., 2011). Second, PEPCk may act in reverse direction from phosphoenolpyruvate to oxaloacetate. This hypothesis is rather unlikely as this reaction directionality would couple anaplerotic carboxylation to the substrate level phosphorylation, generating one GTP molecule. Since the reaction from oxaloacetate to phosphoenolpyruvate is coupled to the hydrolysis of one GTP molecule, it can be expected to be favored. A third alternative represents the glyoxylate shunt, which exclusively fulfills the anaplerotic function in *C. glutamicum* under growth on acetate (Wendisch et al., 1997).

### A Closer Look Into Carbon Balancing

Interestingly, the *C. glutamicum* Δ*ppc* Δ*pyc* mutant also showed an altered ratio of specific glucose uptake and growth rate in comparison to other mutants and the WT (Figure 2A). The observation that less biomass was formed per unit uptake rate raised the question as to where the excess carbon atoms end up. To answer this question, a carbon balance was performed. Contrary to the conventional carbon balancing approach, consisting in the quantification of the total carbon recovery in biomass and exhaust gas by the time all substrate has been consumed (Buchholz et al., 2014), we calculated the instantaneous carbon balance Θ as:

(5) $$\begin{array}{l} \Theta =\frac{\frac{\mu \cdot \omega_{C}}{M_{C}}+\pi_{CO_{2}}}{\pi_{GLC}\cdot 6} \end{array}$$

where μ denotes the specific growth rate given in h^−1^ (here assumed to be constant for the considered exponentially growing cells), ω_*C*_ denotes the mass fraction of carbon in the biomass in g_C_ ${\text{g}}_{\text{CDW}}^{-1}$, *M*_*C*_ denotes the molecular weight of carbon in g mmol^−1^, and π_*GLC*_ as well as π_*CO*_2__ denote the specific d-glucose uptake and carbon dioxide formation rates as derived from Equations (2) to (4).

Equation (5) balances the specific rates of carbon uptake and carbon flow into sinks at any given time. Here biomass and CO_2_ formation are the only considered carbon sinks (any other by-product formation could be excluded for all strains under investigation). The quantity ω_*C*_ has been reported several times in the literature: Marx et al. (1996) reported 0.408 g_C_ ${\text{g}}_{\text{CDW}}^{-1}$ for *C. glutamicum* MH20-22B, a l-lysine producer strain, determined in freeze-dried biomass with a CHNS elemental analyzer. More recently, Buchholz et al. (2014) reported a value of 0.514 ${\text{g}}_{\text{C}}\;{\text{g}}_{\text{CDW}}^{-1}$ for *C. glutamicum* ATCC 13032, which was determined by separately quantifying the total carbon in liquid bioreactor samples (supernatant and biomass) and the total inorganic carbon (total dissolved carbon) in the supernatant. In this work, we experimentally quantified ω_*C*_ for wild-type *C. glutamicum* to be around 0.4–0.42 ${\text{g}}_{\text{C}}\;{\text{g}}_{\text{CDW}}^{-1}$. The uncertainty of this parameter notwithstanding, its value was assumed to be 0.45 ${\text{g}}_{\text{C}}\;{\text{g}}_{\text{CDW}}^{-1}$ for all following calculations. In face of the apparent uncertainty, its standard error was assumed to be 0.05 ${\text{g}}_{\text{C}}\;{\text{g}}_{\text{CDW}}^{-1}$. The arising interval covers all available literature information on this quantity. The uncertainty in derived quantities thereof can be computed by Gaussian error propagation.

From Figure 2B it becomes apparent that the evolved Δ*ppc* Δ*pyc* strain grows with a higher relative CO_2_-formation rate with respect to the uptake rate. At the same time, the sum of the specific rates at which carbon flows into sinks amounts to the same relative value with respect to the carbon uptake rate as in other strains. Therefore, it seems plausible that a higher decarboxylation activity explains the lower relative growth rate in the *C. glutamicum* Δ*ppc* Δ*pyc* mutant. This higher relative CO_2_-formation rate further substantiates the hypothesis that an altered ratio of ICL and ICD activity involves the glyoxylate shunt as anaplerotic reaction sequence in this mutant. Exclusive anaplerotic activity through the glyoxylate shunt would release two equivalents CO_2_ per C4-body of oxaloacetate formed, instead of fixing one CO_2_ as in the case of alternative ME or reversible PEPCk activities.

One result holds true irrespective of the genetic background: The recovery of carbon at any given time in the reactions that act as carbon sinks amounts to 80% of the carbon equivalent of the uptake rate (Figure 2B and Table 2). The non-closed instantaneous carbon balance may hint to extensive by-product formation or indicate systematically biased extracellular rates. The former is unlikely since *C. glutamicum* WT is known to produce only minor by-products under aerobic conditions (the DO was maintained at 30%). Notwithstanding, the genetic alterations may induce a more extensive overflow metabolism in some deletion mutants. However, all organic acids, sugar phosphates and amino acids in the culture supernatant measured by targeted LC-MS/MS account for a total of 274–786 μmol_C_ ${\text{g}}_{\text{CDW}}^{-1}$, depending on the strain. Therefore, the exometabolome can be neglected as carbon sink since the gap in the balance of specific rates amounts to several mmol_C_ ${\text{g}}_{\text{CDW}}^{-1}$ h^−1^ (Figure 2B).

Remarkably, the value of 80% for the instantaneous carbon balance matches the determined carbon balance closure in the study of Buchholz et al. (2014). This study convincingly showed that the gap in carbon balance can be traced back to the fact that part of the CO_2_-production at any given time dissolves as ${\text{HCO}}_{3}^{-}$ in the culture broth. This share is not recovered as gaseous CO_2_ at the detector, leading to an underestimation of the CO_2_-production rate.

### Proteomic and Metabolomic Responses to Gene Deletions in Anaplerotic Reactions

To gain further insight into the metabolism of each mutant, untargeted proteome and targeted metabolome analyses were performed. To ensure comparability, eight strains were cultivated in parallel and subjected to identical and isochronous sample processing in subsequent steps (see section Materials and Methods for detailed descriptions). In total, we analyzed 1199 cytosolic proteins and 48 metabolites of central metabolism.

Since the Δ*ppc* mutant showed no altered phenotype and has been thoroughly characterized before, it was omitted from the set of strains to be analyzed. The evolved strain *C. glutamicum* Δ*ppc* Δ*pyc* showed significant changes in specific proteins and metabolites, which will be discussed separately in the next section.

**Figure 3:**
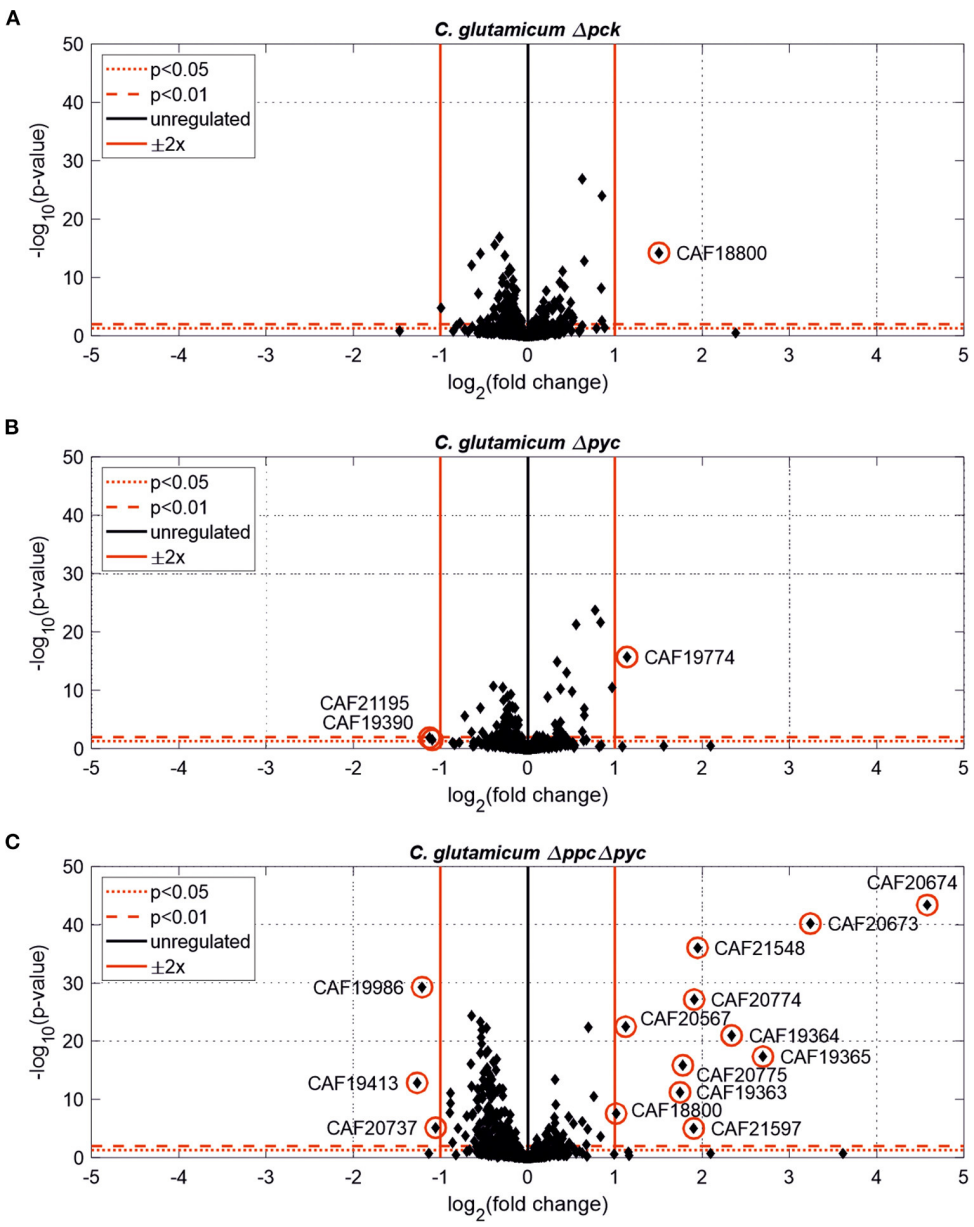
Estimated protein fold-changes for selected *C. glutamicum* deletion mutants in comparison to the wild type. **(A)** *C. glutamicum* Δ*pck*. **(B)** *C. glutamicum* Δ*pyc*. **(C)** *C. glutamicum* Δ*ppc* Δ*pyc*. Volcano plots with significantly changed proteins [*p* < 0.05, |Log2(fold change)| ≥ 0.5] highlighted in red.

Further differentially expressed proteins were found in *C. glutamicum* Δ*pck* and *C. glutamicum* Δ*pyc* (Figure 3). In none of the other tested deletion mutants significantly changed protein abundances [*p* < 0.05, |Log2(fold change)| ≥ 0.5] were found (data not shown).

Only one protein encoded by *cybD* (cg0282) and which might be involved in stress response was found to be up-regulated in the *C. glutamicum* Δ*pck* mutant (Table 3). In *C. glutamicum* Δ*pyc* the enzyme quinolinate synthase A encoded by the *nadA* gene (cg1216) was up-regulated. This enzyme catalyzes the condensation of iminoaspartate with dihydroxyacetone phosphate to form quinolinate, and represents the second step of the *de novo* synthesis of NAD^+^. The latter starts from l-aspartate and up-regulation of this enzyme could be a cellular response to the limited availability of this amino acid following the inactivation of PCx and to ensure sufficient NAD^+^ supply. In addition, the putative transcriptional regulator (cg0787) and the 50S ribosomal protein L36 encoded by the *rpmJ* gene (cg2791) were found to be down-regulated in *C. glutamicum* Δ*pyc*.

**Table 3:** Differentially expressed proteins of *C. glutamicum* anaplerotic deletion mutants in comparison to the wild-type strain.

**Strain**	**Protein ID**	**Cg no**.	**Gene**	**Annotated function**	**Fold change**	***p*-value**
*C. glutamicum* Δ*pck*	CAF18800	cg0282	*cybD*	Putative protein, CsbD-family, probably involved in stress response	2.84	5.90e-15
*C. glutamicum* Δ*pyc*	CAF19774	cg1216	*nadA*	Quinolinate synthase A	2.20	2.03e-16
	CAF19390	cg0787	–	Transcriptional regulator	0.47	3.11e-02
	CAF21195	cg2791	*rpmJ*	50S ribosomal protein L36	0.46	1.40e-02
*C. glutamicum* Δ*ppc* Δ*pyc*	CAF20674	cg2560	*aceA*	Isocitrate lyase	23.94	3.79e-44
	CAF20673	cg2559	*aceB*	Malate synthase	9.45	6.63e-41
	CAF19365	cg0762	*prpC2*	2-methylcitrate synthase	6.49	4.73e-18
	CAF19364	cg0760	*prpB2*	2-methylcitrate lyase	5.05	1.07e-21
	CAF21548	cg1737	*acn*	Aconitase	3.86	8.53e-37
	CAF20774	cg3047	*ackA*	Acetate kinase	3.75	7.39e-28
	CAF21597	cg1792	*whiA*	Putative transcriptional regulator-WhiA homolog	3.75	9.38e-06
	CAF20775	cg3048	*pta*	Phosphate acetyltransferase	3.43	1.42e-16
	CAF19363	cg0759	*prpD2*	2-methylcitrate dehydratase	3.36	5.83e-12
	CAF20567	–	–	Hypothetical protein	2.18	3.42e-23
	CAF18800	cg0282	–	Conserved hypothetical protein	2.02	2.79e-08
	CAF20737	cg3008	*porA*	Porin	0.48	7.16e-06
	CAF19986	cg1451	*serA*	Phosphoglycerate dehydrogenase	0.43	5.03e-30
	CAF19413	cg0812	*dtsR1*	Acetyl/ propionyl-CoA carboxylase beta chain	0.42	1.34e-13

*All proteins with significant changes [p < 0.05, Log2(fold change) > 0.5 for upregulated proteins and Log2(fold change) < −0.5 for down-regulated proteins] are listed*.

**Figure 4:**
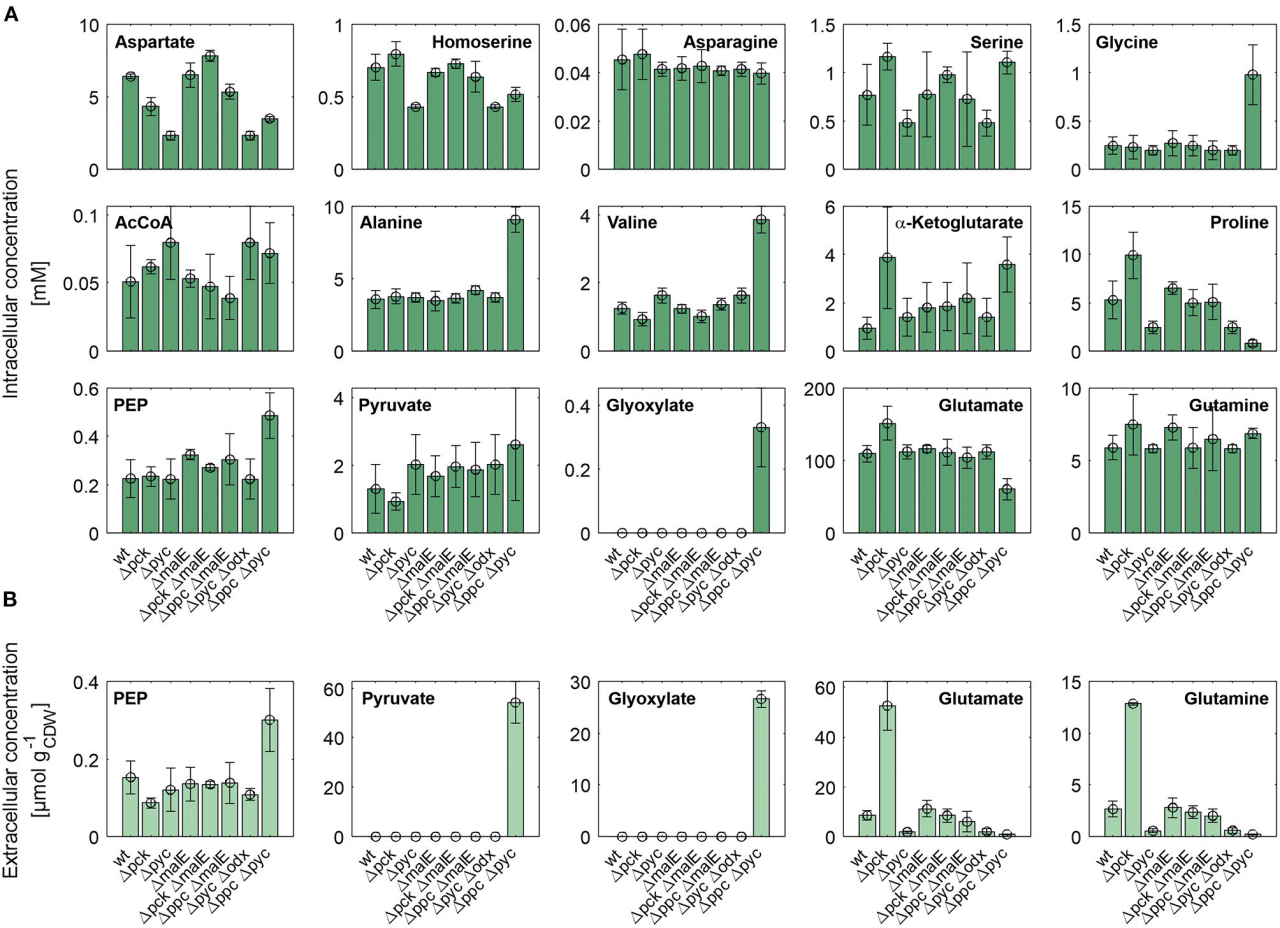
Intracellular **(A)** and specific extracellular **(B)** concentrations of selected metabolites in *C. glutamicum* wild type and anaplerotic deletion mutants cultivated under controlled bioreactor conditions in CGXII medium with d-glucose as sole carbon and energy source. For gene to protein references see Figure 2.

Figure 4 shows intracellular and extracellular concentrations of selected metabolites. The l-aspartate pool is most closely correlated with the growth rate, i.e., the lowest concentrations were observed for all mutants with reduced growth rate. Apparently, its supply appears to be limiting the growth as the restored growth rate of the Δ*pck* Δ*malE* mutant in comparison to the single deletion strain Δ*pck* goes hand in hand with increased l-aspartate supply. The concentration pattern of l-aspartate seems to reflect itself in the l-homoserine pool, which is derived from the former through three intermediate reaction steps, consuming two NAD(P)H molecules and one ATP molecule. In contradistinction to l-aspartate, however, l-homoserine is clearly higher concentrated in the Δ*pck* mutant. Since the reaction sequence between both intermediates is redox-dependent, one may be tempted to attribute this observation to redox balancing. The biosynthesis of l-glutamate from α-ketoglutarate and of l-proline from l-glutamate are also redox-dependent. Conspicuously, both pools are also higher concentrated in this strain (Figure 4).

Moreover, we analyzed intracellular levels of NADPH and NADH alongside their oxidized analogs. No significant difference was detected in the reduced forms of these co-factors (data not shown). However, the mean of relative standard deviation over all mutants for NADH and NADPH concentrations amounts to 46% and 48%, respectively. This high technical error, impeding a precise quantification, cannot be traced back to inaccuracies in pipetting or BV concentration measurements since these factors also apply to all other metabolite quantifications in the same sample. Since the mean relative standard deviation for other metabolite pools was below 10% these factors appear to have been controlled quite well. The most likely reason for the observed coefficients of variation is metabolite instability. The redox equivalents are known to be quite sensitive to oxidation and degradation (Siegel et al., 2014). Slightly different temperature time courses, residual enzymatic activity in metabolite extracts and oxidation most likely account for the observed differences. Therefore, no accurate conclusion about the redox state in each mutant could be drawn. Nonetheless, the fact that the removal of a redox-dependent enzyme like ME restores the growth rate of the *C. glutamicum* Δ*pck* mutant suggests an involvement of the redox balance mediating some of the observed changes in metabolite pools.

### Glyoxylate Shunt Enables Growth of *C. glutamicum* Δ*ppc* Δ*pyc* on d-Glucose as Sole Carbon and Energy Source

Whole-genome sequencing of evolved *C. glutamicum* Δ*ppc* Δ*pyc* confirmed the absence of genes *ppc* and *pyc* across the cell population (Table 4), excluding any growth contamination effect. Two insertions and some SNPs (see Supplementary Table 6) were detected in the coding region for ICD. Moreover, ICL (cg2560), MS (cg2559), and *cis*-aconitase (ACN, cg1737) are highly up-regulated in *C. glutamicum* Δ*ppc* Δ*pyc* (Figure 3 and Table 3) and only in this mutant intracellular and extracellular accumulation of glyoxylate was detected (Figure 4).

**Table 4:** Structural variants identified in the *C. glutamicum* Δ*ppc* Δ*pyc* mutant adapted to d-glucose as sole carbon source in comparison to *C. glutamicum* WT as reference.

**Affected region**	**Character**	**nt Region**	**Length**	**Reads of position**	**Rel. frequency**
In cg2353, putative protein	Deletion	2,236,684.2,238,317	1,634	47	1
In cg2854, *tnp2c*, transposase	Deletion	2,716,280.2,717,915	1,636	59	1
In cg0691, *groEL*′, 60 kDa chaperonin, N-terminal fragment	Deletion	610,994.612,446	1,453	85	0.99
In cg0791, *pyc*, pyruvate carboxylase	Deletion	707,185.709,613	2,429	76	0.99
In cg1787, *ppc*, phosphoenolpyruvate carboxylase	Deletion	1,679,298.1,681,219	1,922	60	0.98
Intergenic region of cg1860 (putative membrane protein) and cg1861 (*rel*, ppGpp synthetase / ppGpp pyro-phosphorylase)	Replacement	1,754,002.1,754,038	37	73	0.98
Upstream of cg0756, *cstA*, carbon starvation protein A	Deletion	669,585.669,597	13	46	0.9
In cg2262, *ftsY*, signal recognition particle GTPase	Deletion	2,144,947.2,144,964	18	13	0.4
In cg0766, *icd*, isocitrate dehydrogenase	Insertion	681,078.681,079	57	27	0.38
In cg0766, *icd*, isocitrate dehydrogenase	Insertion	681,136.681,137	57	26	0.32
In cg0953, *mctC*, monocarboxylic acid transporter	Deletion	884,479.884,487	9	13	0.31

*The column “Reads of position” refers to the number of sequencing reads supporting the alteration. The relative frequency refers to the number of reads supporting the alteration relative to the total number of reads of this position or region*.

These findings are in agreement with the study of Schwentner et al. and point to a redirection of carbon flux in our evolved strain from the oxidative decarboxylation branch of the TCA cycle into the glyoxylate shunt (Schwentner et al., 2018). In the absence of C3-carboxylation activity at the anaplerotic node, accumulation of the substrate pools phosphoenolpyruvate and pyruvate can be expected. Indeed, phosphoenolpyruvate was significantly higher concentrated in the evolved Δ*ppc* Δ*pyc* strain, while the intracellular pyruvate pool remained unaffected (Figure 4). However, metabolite levels of l-alanine and l-valine, which are directly derived from pyruvate, were strongly increased and this finding is also consistent with previous data (Schwentner et al., 2018). The lack of statistical significance of strain differences in the pyruvate pool is most likely due to higher technical errors during metabolite quantification. It is well-established that the accurate quantification of organic acids in cell extracts represents a veritable challenge (Zimmermann et al., 2014).

Moreover, the metabolite pool of l-glycine shows one of the most significant concentration changes, clearly distinguishing the evolved Δ*ppc* Δ*pyc* strain. l-glycine, in turn, is derived from l-serine, which also showed an increased concentration (Figure 4). It appears that the missing carboxylation rate cannot be matched by the pyruvate dehydrogenase activity in the Δ*ppc* Δ*pyc* mutant for substrate pools like phosphoenolpyruvate as well as amino acids derived from the lower glycolytic intermediates appear to accumulate intracellularly. This would also explain why the phosphoglycerate dehydrogenase encoded by *serA* (cg1451) was found to be significantly down-regulated in this mutant (Table 3). The enzyme catalyzes the first step in the biosynthesis of l-glycine, l-serine and l-cysteine and its down-regulation could be the cellular response to the higher availability of 3-phosphoglycerate.

In terms of glyoxylate shunt regulation, Wendisch et al. suggested that the carbon-source dependent regulation of this pathway is mediated by intracellular acetyl-CoA concentrations (Wendisch et al., 1997). However, intracellular acetyl-CoA concentrations did not vary significantly with respect to strain background (Figure 4), and therefore this hypothesis could not be validated with the made intracellular measurements. Unfortunately, acetyl-CoA measurements are notoriously error-prone due to the high instability of thioesthers. Though taking strenuous efforts to keep the sample below−20°C and immediate analysis after extraction, we still obtained a highly variable signal within each treatment group.

The corresponding genes *aceA* and *aceB* of ICL and MS, respectively, are thought to be repressed by the regulator protein RamB (cg0444) under glycolytic conditions (Auchter et al., 2011). Moreover, it has been established that RamA (cg2831) acts as transcriptional activator of both genes when acetate is the carbon source. However, no significant change in the abundances of RamA and RamB were found in Δ*ppc* Δ*pyc* mutant compared to the wild type. The fact that a Δ*ramA* mutant is not able to grow on acetate suggests that RamA is strictly required as transcriptional activator for increased expression of at least one of the essential enzymes of acetate assimilation, which are acetate kinase (AK) encoded by *ackA* (cg3047), phosphotransacetylase (PTA) encoded by *pta* (cg3048), ICL and MS (Cramer and Eikmanns, 2007). Indeed, AK and PTA are also significantly up-regulated in the Δ*ppc* Δ*pyc* mutant (Table 3).

Most interestingly, this strain also shows an up-regulation of the three enzymes of the methylcitrate cycle in *C. glutamicum* (Claes et al., 2002), namely 2-methylcitrate synthase (PrpC2) encoded by *prpC2* (cg0762), 2-methylcitrate dehydratase (PrpD2) encoded by *prpD2* (cg0759) and 2-methylcitrate lyase (PrpB2) encoded by *prpB2* (cg0760) (Table 3). Together with the activities of AK and PTA this cycle is known to be the predominant route for the degradation of propionate into pyruvate and succinate (Figure 1). In the presence of propionate all three genes are transcriptionally activated by the regulator PrpR (Plassmeier et al., 2012). Under the applied d-glucose conditions, however, no change in the PrpR abundance was detected. Moreover, it was found that the *prpDBC2* operon is transcriptionally activated by RamA, but not affected by RamB (Auchter et al., 2011). This makes the conclusion compelling that the concentration of at least one, still unknown, metabolite effector binding to RamA was altered, leading to the activation of the aforementioned genes. Following the strong up-regulation of the *prpDBC2* cluster in combination with the glyoxylate shunt it might be speculated that the surplus of pyruvate (from PEPCx and PCx inactivation) and succinate (from ICL activity) is channeled into the methylcitrate cycle, operating in the reverse direction. This route would represent a by-pass of the TCA cycle reactions succinate: quinone oxidoreductase (SQO), fumarase (FUM) and malate: quinone oxidoreductase (MQO) to provide oxaloacetate as biomass precursor (Figure 1). From a thermodynamic point of view, the PrpB2 reaction in direction of 2-methylisocitrate is favored (Δ_*r*_*G*^′0^ = −9.3 *kJ/mol*) in comparison to the SQO reaction leading to fumarate (Δ_*r*_*G*^′0^ = 14.5 *kJ/mol*). However, the potentially last step of the methylcitrate cycle catalyzed by PrpC2 is thermodynamically very unfavorable (Δ_*r*_*G*^′0^ = 39.2 *kJ/mol*), but might still work under *in vivo* conditions due to very low concentrations of oxaloacetate (<100 nM of lower detection limit of applied LC-QqQ MS). Indeed, Plassmeier et al. showed that the *prpDBC2* operon is also expressed under normal cell growth conditions in wild-type *C. glutamicum*, independent of the application of propionate as carbon source (Plassmeier et al., 2007). Moreover, a high up-regulation of the *prpDBC2* operon was found in a *C. glutamicum* strain engineered for overproduction of l-isoleucine (Ma et al., 2018). Here it was speculated that this up-regulation was due to a high intracellular formation of propionyl-CoA, which was derived from the intermediate 2-ketobutyrate of the l-isoleucine biosynthesis. The resulting propionyl-CoA was then further converted into a polyhydroxyalkanoate by heterologous expression of the *phaCAB* gene cluster. In our case, however, we can exclude propionyl-CoA formation from 2-ketobutyrate because l-aspartate is limiting. Additionally, it remains open what happens with propionyl-CoA as the second product of the PrpC2 reaction when running in reverse direction (Figure 1). Therefore, further investigations on the interplay between the different pathways are required to substantiate our hypothesis.

Moreover, we found a SNP in the intergenic region between cg3314 and cg3315, four nucleotides upstream from the translation start of MalR (*malR*, cg3315). This protein has been originally identified as repressor of the *malE* gene in the study of Krause et al. (2012) and was recently reported to bind to several other loci (Hünnefeld et al., 2019). Since the mutation occurred outside the coding sequence of this regulator, only expression changes of the gene are able to affect metabolism. Unfortunately, the specific protein data for MalR was not very accurate (*p* > 0.5) to enable any conclusion, but ME appeared to be down-regulated in the evolved Δ*ppc* Δ*pyc* strain when grown on d-glucose as sole carbon and energy source (0.54-fold, *p* < 4.73e-10). Since purified ME of *C. glutamicum* was shown to carboxylate pyruvate in *in vitro* assays with an apparent *K*_m_ constant of 13.8 mM (Gourdon et al., 2000) it was speculated that ME serves as anaplerotic enzyme under circumstances when PEPCx and PCx activities are absent and pyruvate availability is still ensured through running glycolysis. In our case, however, the amount of ME was significantly reduced with respect to the wild type and its intracellular substrate pool of pyruvate is not significantly altered and well below the *K*_m_ value (Figure 4A). Therefore, it can be excluded that ME catalyzes a flux compensating for the missing PCx and PEPCx activity in the evolved Δ*ppc* Δ*pyc* strain.

Finally, a deletion was detected in the *mctC* gene (cg0953), which has been shown to be essential in *C. glutamicum* for the uptake of pyruvate (Jolkver et al., 2009). Based on this, it can be hypothesized that the genetic alteration in this transporter may lead to a decreased uptake of pyruvate from the medium, which would explain the extracellular accumulation of pyruvate exclusively found in the Δ*ppc* Δ*pyc* mutant (Figure 4B).

## Conclusions

We characterized eight different mutant strains of *C. glutamicum* carrying single or double deletions in five anaplerotic enzymes. The metabolism and adaptation of each mutant during growth under defined d-glucose conditions in lab-scale bioreactors was investigated by quantification of its extracellular rates, central metabolic intermediates by LC-QqQ MS and proteome by SWATH acquisition using a LC-QqTOF MS platform.

In comparison to the wild type the four deletion mutants *C. glutamiucm* Δ*pyc, C. glutamiucm* Δ*pyc* Δ*odx, C. glutamiucm* Δ*ppc* Δ*pyc*, and *C. glutamiucm* Δ*pck* showed lowered specific growth rates and d-glucose uptake rates, underlining the importance of PCx and PEPCk activity for a balanced carbon and energy flux at the anaplerotic node.

Detailed analyses of the *C. glutamicum* Δ*ppc* Δ*pyc* mutant evolved to grow on d-glucose revealed the strong up-regulation of a few genes that are under control of the transcriptional regulator RamA. Higher protein abundances were found for the enzymes of the glyxoylate shunt as well as the methylcitrate cycle under solely glycolytic conditions, under which condition the corresponding genes were thought to be repressed. It is inferred that this adaptation must be due to a changed concentration of a metabolite affecting the activity of regulator protein RamA, brought about by a concentration change of the former. This metabolite pool, however, could not be identified from the set of metabolites from glycolysis, TCA cycle and amino acids that was targeted in this study. Since the encoding genes of the altered proteins represent just a small subset of the RamA regulon, it can be concluded that the binding affinities of RamA to all target genes may be regulated by various effector metabolites and not a single one. Further research, especially based on untargeted metabolomics, will be needed to identify the metabolite regulator(s) active on RamA under d-glucose conditions.

In conjunction with the intracellular metabolomics data we generally conclude that *C. glutamicum* is able to compensate missing carboxylation activities of PEPCx and PCx by activation of the glyoxylate shunt, potentially in combination with the methylcitrate cycle to channel the higher levels of PEP/ pyruvate as well as succinate and thereby also contributing to replenish oxaloacetate. To further substantiate the hypothesis on the reverse operation of the methylcitrate cycle isotope-based metabolic flux analyses with the evolved *C. glutamicum* Δ*ppc* Δ*pyc* strain could be conducted in further studies.

Finally, the reproducible effect of bicarbonate formation under excess d-glucose conditions and its consequences for carbon balancing also requires further investigations. For example, a combination of batch experiments under variation of pH and gassing rate as well as thorough modeling of the resulting CO_2_-dynamics in the gas and liquid phase could be an approach for a more accurate determination of CO_2_-formation rates.

## Data Availability Statement

The mass spectrometry proteomics data have been deposited to the ProteomeXchange Consortium via the PRIDE (Perez-Riverol et al., 2019) partner repository with the dataset identifier PXD022622. The reads data of the Δ*ppc* Δ*pyc* strain are available in NCBI's SRA via BioProject ID PRJNA678589. The other datasets generated for this study are available on request to the corresponding author.

## Author Contributions

JK and SN designed the research. JK performed data analysis and wrote the manuscript. JK and MP performed the bioreactor cultivations of *C. glutamicum*. BK lysed and digested all samples and performed the LC-MS/MS measurements. JL constructed the *C. glutamicum* double deletion mutants used in this manuscript. TP performed the whole-genome sequencing. SN, TP, and WW revised the manuscript. SN, RT, and BB supervised the research. All authors have given approval to the final version of the manuscript.

## Conflict of Interest

The authors declare that the research was conducted in the absence of any commercial or financial relationships that could be construed as a potential conflict of interest.
