# Supplementary material for: Comprehensive Analysis of C. glutamicum Anaplerotic Deletion Mutants Under Defined d-Glucose Conditions
Source: Front Bioeng Biotechnol. 2021 Jan 20;8:602936. doi: 10.3389/fbioe.2020.602936 (PMC7855459; doi:10.3389/fbioe.2020.602936)
Supplement: Supplementary file 1 [file Data_Sheet_1.PDF]

Supplementary Material

Comprehensive analysis of *C. glutamicum* anaplerotic deletion mutants under defined D-glucose conditions

Jannick Kappelmann[1], Bianca Klein[1], Mathias Papenfuß[2], Julian Lange[3], Bastian Blombach[4], Ralf Takors[3], Wolfgang Wiechert[1], Tino Polen[1], Stephan Noack[1,*]

Supplementary Table S1. Estimated specific growth rates (μ), D-glucose consumption rates (π_{GLC}), CO_2 formation rates (π_{CO2}) and instantaneous carbon balance (θ) for anaplerotic deletion mutants during exponential growth.

Strain	Replicate	μ [h^{-1}] (BV)	μ [h^{-1}] (CDW)	π_{GLC} [mmol mL^{-1} h^{-1}] (BV)	π_{GLC} [mmol g_{CDW}^{-1} h^{-1}] (CDW)	π_{CO2} [mmol mL^{-1} h^{-1}] (BV)	π_{CO2} [mmol g_{CDW}^{-1} h^{-1}] (CDW)	Θ [-]
ATCC 13032	R1	0.45 [0.44, 0.46]	0.44 [0.42, 0.45]	1.47 [1.45, 1.50]	4.86 [4.68, 5.05]	2.20	7.16 ± 0.60	0.81 ± 0.077
ATCC 13032	R2	0.45 [0.44, 0.46]	0.45 [0.42, 0.48]	1.41 [1.36, 1.46]	4.61 [4.40, 4.85]	1.92	6.41 ± 0.67	0.84 ± 0.087
ATCC 13032	R3	0.47 [0.45, 0.48]	0.46 [0.42, 0.49]	1.53 [1.48, 1.58]	4.98 [4.77, 5.23]	2.23	7.24 ± 0.71	0.82 ± 0.089
Δ*pck*	R1	0.36 [0.36, 0.37]	0.37 [0.37, 0.38]	1.26 [1.25, 1.27]	4.03 [3.93, 4.14]	1.63	5.32 ± 0.51	0.80 ± 0.072
Δ*pck*	R2	0.38 [0.37, 0.38]	0.39 [0.37, 0.40]	1.27 [1.25, 1.29]	4.16 [4.02, 4.29]	1.75	5.69 ± 0.53	0.81 ± 0.077
Δ*ppc*	R1	0.45 [0.44, 0.47]	0.46 [0.42, 0.49]	1.57 [1.53, 1.61]	5.08 [4.89, 5.32]	2.43	7.60 ± 0.92	0.81 ± 0.083
Δ*ppc*	R2	0.43 [0.42, 0.44]	0.43 [0.41, 0.40]	1.48 [1.43, 1.53]	5.16 [4.95, 5.38]	2.36	7.68 ± 1.09	0.77 ± 0.076
Δ*pyc*	R1	0.38 [0.37, 0.39]	0.40 [0.38, 0.41]	1.44 [1.41, 1.46]	4.60 [4.45, 4.77]	2.24	7.29 ± 0.51	0.80 ± 0.072
Δ*pyc*	R2	0.37 [0.36, 0.37]	0.40 [0.38, 0.41]	1.55 [1.53, 1.57]	4.70 [4.57, 4.84]	2.32	7.56 ± 0.76	0.79 ± 0.071
Δ*malE*	R1	0.48 [0.48, 0.49]	0.46 [0.45, 0.48]	1.50 [1.47, 1.52]	5.04 [4.98, 5.21]	2.02	7.36 ± 0.43	0.82 ± 0.075
Δ*malE*	R2	0.49 [0.48, 0.50]	0.46 [0.44, 0.48]	1.37 [1.36, 1.39]	4.72 [4.58, 4.87]	1.82	6.64 ± 1.00	0.85 ± 0.080
Δ*pck* Δ*malE*	R1	0.45 [0.43, 0.47]	0.45 [0.41, 0.48]	1.50 [1.45, 1.54]	4.94 [4.72, 5.18]	2.30	7.45 ± 0.67	0.82 ± 0.087
Δ*pck* Δ*malE*	R2	0.47 [0.46, 0.48]	0.47 [0.45, 0.48]	1.48 [1.44, 1.52]	4.77 [4.58, 4.99]	2.26	7.37 ± 0.31	0.87 ± 0.083
Δ*pck* Δ*malE*	R3	0.43 [0.43, 0.44]	0.45 [0.43, 0.47]	1.51 [1.49, 1.52]	4.85 [4.69, 5.02]	2.15	6.98 ± 0.83	0.81 ± 0.076
Δ*ppc* Δ*malE*	R1	0.46 [0.45, 0.47]	0.43 [0.42, 0.44]	1.46 [1.43, 1.49]	5.03 [4.83, 5.25]	2.14	6.96 ± 0.29	0.77 ± 0.072
Δ*ppc* Δ*malE*	R2	0.42 [0.41, 0.42]	0.42 [0.41, 0.43]	1.48 [1.45, 1.51]	4.77 [4.57, 4.96]	1.89	6.16 ± 0.83	0.77 ± 0.073
Δ*ppc* Δ*malE*	R3	0.43 [0.42, 0.45]	0.43 [0.41, 0.46]	1.40 [1.35, 1.45]	4.73 [4.48, 4.96]	2.23	7.25 ± 1.92	0.83 ± 0.088
Δ*pyc* Δ*odx*	R1	0.39 [0.38, 0.40]	0.40 [0.38, 0.42]	1.40 [1.38, 1.43]	4.54 [4.37, 4.70]	2.00	6.51 ± 0.96	0.78 ± 0.077
Δ*pyc* Δ*odx*	R2	0.38 [0.38, 0.38]	0.38 [0.37, 0.39]	1.31 [1.30, 1.32]	4.15 [4.04, 4.28]	1.87	6.07 ± 0.99	0.82 ± 0.074
Δ*pyc* Δ*odx*	R3	0.43 [0.42, 0.44]	0.42 [0.40, 0.44]	1.26 [1.22, 1.30]	4.44 [4.31, 4.60]	1.78	6.36 ± 0.89	0.83 ± 0.078
Δ*ppc* Δ*pyc*	R1	0.27 [0.27, 0.28]	0.27 [0.26, 0.28]	1.09 [1.06, 1.11]	3.62 [3.51, 3.77]	2.30	7.52 ± 0.68	0.81 ± 0.070
Δ*ppc* Δ*pyc*	R2	0.26 [0.26, 0.28]	0.26 [0.25, 0.27]	1.20 [1.18, 1.23]	3.85 [3.69, 4.01]	2.23	7.27 ± 0.45	0.74 ± 0.067

Supplementary Table S2. Gradient elution for amino acid quantification.

**Time** [min]	**Flow rate** [µL min^{-1}]	**% B**
0	400	15
10	400	15
16	400	100
28	400	100
30	400	15
35	400	15

Supplementary Table S3. Gradient elution for sugar and nucleoside phosphate quantification.

**Time** [min]	**Flow rate** [µL min^{-1}]	**% B**
0	450	0
2	450	0
5	450	20
8	450	20
10	450	35
14	450	100
15	450	100
15.5	450	0
17	450	0

Supplementary Table S4. Gradient elution for SWATH acquisition.

**Time** [min]	**Flow rate** [µL min^{-1}]	**% B**
-12	200	3
0	200	3
70	200	40
78	200	40
79	200	60
89	200	60
90	200	3

Supplementary Table S5. SWATH Q1 windows monitored during elution on the TripleTOF 6600. The SWATH windows were not scheduled. CES, collision energy spread was always fixed to 5.000.

**SWATH Exp Index**	**StartMass** [Da]	**StopMass** [Da]
SWATH Exp 1	199.50	222.10
SWATH Exp 2	221.10	242.80
SWATH Exp 3	241.80	264.40
SWATH Exp 4	263.40	280.60
SWATH Exp 5	279.60	295.00
SWATH Exp 6	294.00	307.60
SWATH Exp 7	306.60	319.30
SWATH Exp 8	318.30	330.10
SWATH Exp 9	329.10	340.00
SWATH Exp 10	339.00	349.90
SWATH Exp 11	348.90	358.90
SWATH Exp 12	357.90	367.90
SWATH Exp 13	366.90	376.00
SWATH Exp 14	375.00	385.00
SWATH Exp 15	384.00	393.10
SWATH Exp 16	392.10	401.20
SWATH Exp 17	400.20	410.20
SWATH Exp 18	409.20	419.20
SWATH Exp 19	418.20	428.20
SWATH Exp 20	427.20	438.10
SWATH Exp 21	437.10	448.90
SWATH Exp 22	447.90	459.70
SWATH Exp 23	458.70	472.30
SWATH Exp 24	471.30	485.80
SWATH Exp 25	484.80	500.20
SWATH Exp 26	499.20	517.30
SWATH Exp 27	516.30	537.10
SWATH Exp 28	536.10	559.60
SWATH Exp 29	558.60	584.80
SWATH Exp 30	583.80	613.60
SWATH Exp 31	612.60	645.10
SWATH Exp 32	644.10	679.30
SWATH Exp 33	678.30	718.90
SWATH Exp 34	717.90	766.60
SWATH Exp 35	765.60	826.00
SWATH Exp 36	825.00	903.40
SWATH Exp 37	902.40	1013.20
SWATH Exp 38	1012.20	1171.60
SWATH Exp 39	1170.60	1419.10
SWATH Exp 40	1418.10	1998.70

Supplementary Table S6. SNPs or affected amino acids in intergenic regions or protein-coding genes identified in the *C. glutamicum* Δ*ppc* Δ*pyc* mutant adapted to D-glucose as sole carbon source in comparison to *C. glutamicum* WT as reference. The column 'Reads of position' refers to the number of sequencing reads supporting the alteration. The relative frequency refers to the number of reads supporting the alteration relative to the total number of reads of the position.

**Affected region**	**Reads of position**	**Rel. frequency**
nt 3,163,180; SNV C to T, intergenic region of cg3314 (conserved hypothetical protein) and cg3315 (*malR*, transcriptional regulator MalR)	181	1
nt 2,030,620; SNV C to A, intergenic region of cg2136 (*gluA*, glutamate uptake system) and cg2137 (*gluB*, secreted glutamate-binding protein)	125	1
SNV C to T, T306T in cg1574, *pheS*, phenylalanyl-tRNA synthetase α subunit	116	1
SNV T to C, exchange L328S in cg1245, putative membrane protein	102	1
SNV T to C, A22A in cg3237, *sodA* (sod), manganese superoxide dismutase	125	1
SNV A to G, exchange Y158H in cg1676, *lip3*, putative lipase	106	0.99
SNV A to C, exchange E324A in cg1451, *serA*, phosphoglycerate dehydrogenase	75	0.35
SNV C to T, exchange G577S in cg0766, *icd*, isocitrate dehydrogenase	148	0.33
SNV G to A, exchange P583S in cg0766, *icd,* isocitrate dehydrogenase	149	0.14
SNV A to T, Stop L508* in cg2267, putative membrane protein	89	0.11
SNV A to T, exchange V117E in cg0237, putative short chain dehydrogenase	76	0.11

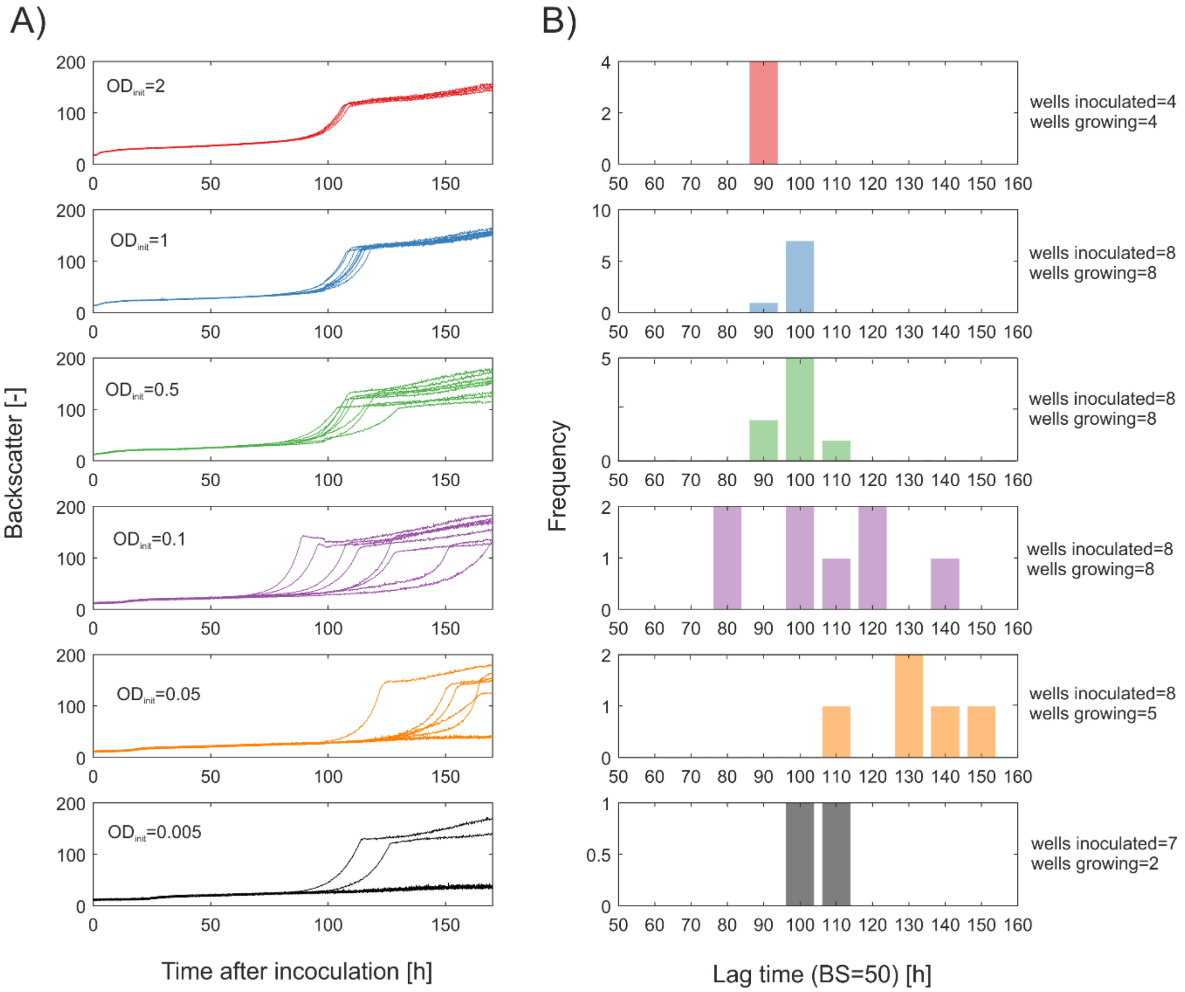

Supplementary Figure S1. Evolution of the *C. glutamicum* Δ*ppc* Δ*pyc* mutant on 1 % D-glucose CGXII medium. A) Time course of biomass growth where each line represents one well of a certain inoculation density. The inoculation density is indicated on the left hand side of each plot, decreasing from top to bottom. B) Histograms of the lag-times in each well of a certain inoculation density. Lag-time is defined as the time after inoculation needed to reach the Backscatter value of 50. In each plot the number of wells is given that were inoculated to the $OD_{init,600}$ indicated under A) alongside the number of wells that reached the Backscatter value of 50.

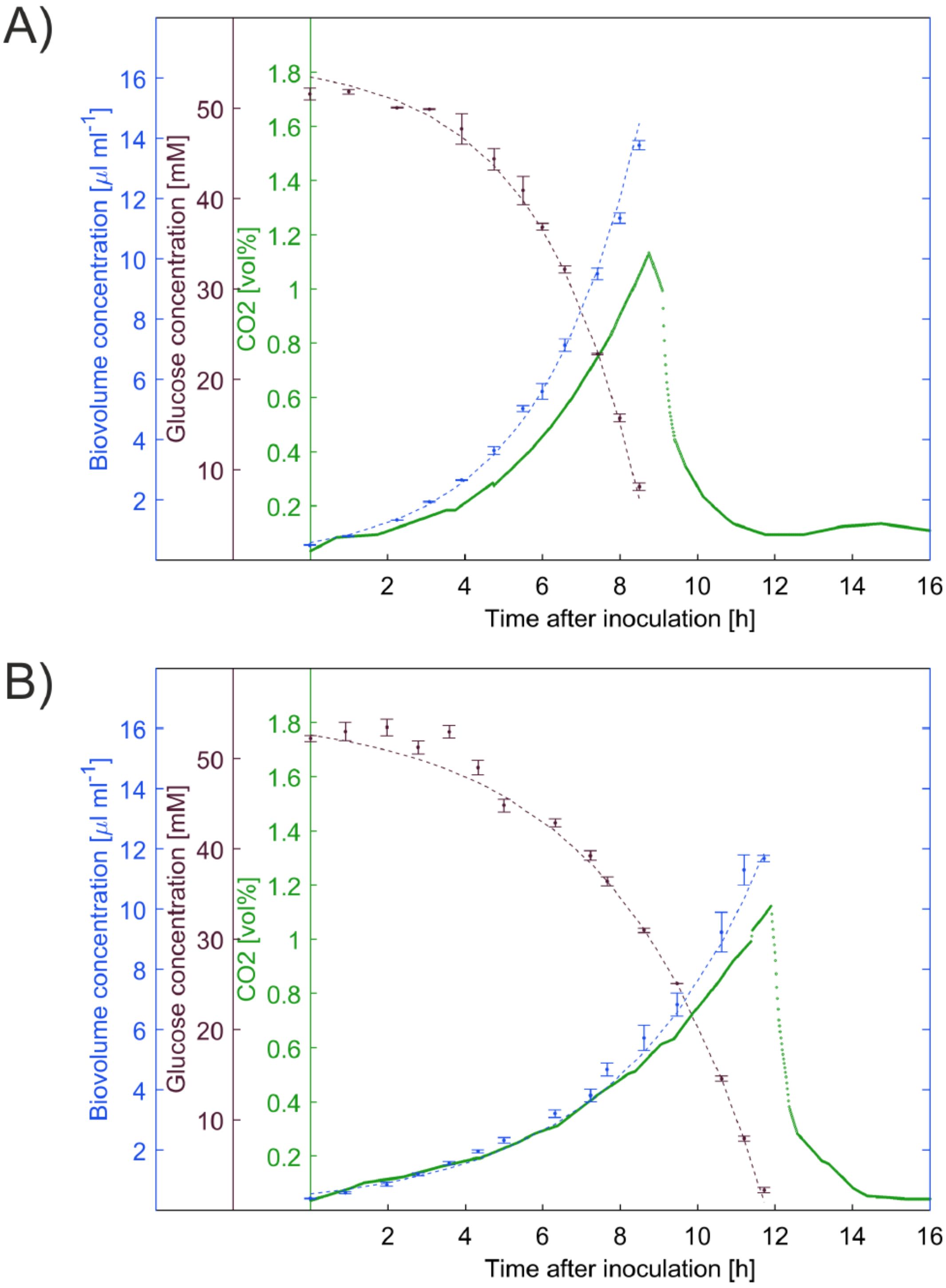

Supplementary Figure S2. Time course of biovolume (BV) and substrate concentration as well as CO_2 exhaust gas volume fraction from a cultivation of the A) Δ*pck* mutant, and B) evolved Δ*ppc* Δ*pyc* mutant on defined CGXII medium with 1% D-glucose. Dashed lines show the computed time course of BV and glucose concentrations for the best fit of Eqs. (2) and (3) in the main text to the shown experimental data. Green filled circles denote the time-dependent volume fraction of CO_2 in the exhaust gas stream.

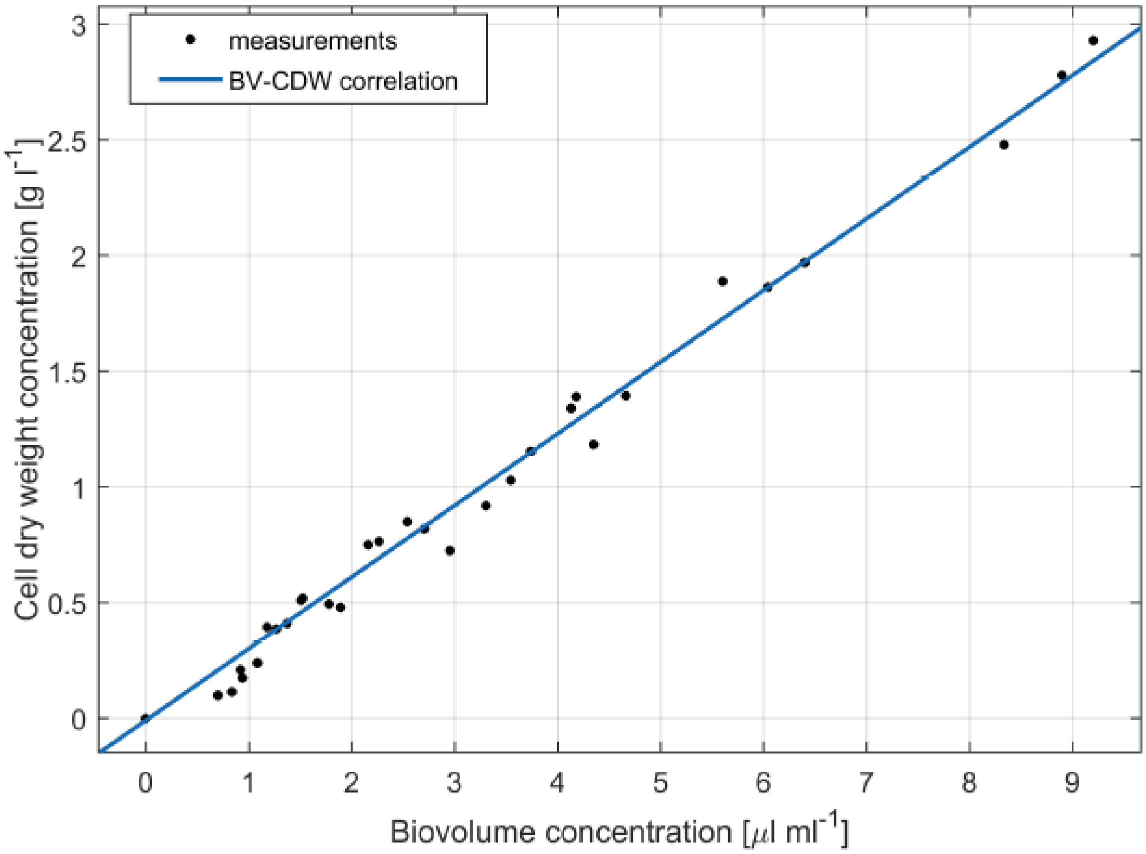

Supplementary Figure S3. Correlation between BV and CDW for *C. glutamicum*. Data were derived by growing the Δ*pyc* Δ*odx*, Δ*ppc* Δ*malE* and wild-type strain in defined CGXII medium with D-glucose as sole carbon and energy source. The fitted coefficients for the function $f(CDW) = p_1 \cdot BV + p_2$ are as follows (coefficient estimate followed by 95 % confidence interval): slope $p_1 = 0.3098\ (0.3045, 0.3151)$ and intercept $p_2 = -0.008692\ (-0.02189, 0.004509)$. The goodness of fit is SSE: 0.2256, R-square: 0.994, Adjusted R-square: 0.9939, RMSE: 0.05278.
